# Potential Applications of Thermophilic Bacteriophages in One Health

**DOI:** 10.3390/ijms24098222

**Published:** 2023-05-04

**Authors:** Hong Liu, Milad Kheirvari, Ebenezer Tumban

**Affiliations:** School of Veterinary Medicine, Texas Tech University, Amarillo, TX 79106, USA

**Keywords:** bacteriophages, thermophilic bacteriophages, lytic enzymes, endolysins, virus-like particles, vaccines, veterinary medicine, food safety, One Health

## Abstract

Bacteriophages have a wide range of applications such as combating antibiotic resistance, preventing food contamination for food safety, and as biomarkers to indirectly assess the quality of water. Additionally, bacteriophage components (endolysins and coat proteins) have a lot of applications in food processing, vaccine design, and the delivery of cargo to the body. Therefore, bacteriophages/components have a multitude of applications in human, plant/veterinary, and environmental health (One Health). Despite their versatility, bacteriophage/component use is mostly limited to temperatures within 4–40 °C. This limits their applications (e.g., in food processing conditions, pasteurization, and vaccine design). Advances in thermophilic bacteriophage research have uncovered novel thermophilic endolysins (e.g., ΦGVE2 amidase and MMPphg) that can be used in food processing and in veterinary medicine. The endolysins are thermostable at temperatures > 65 °C and have broad antimicrobial activities. In addition to thermophilic endolysins, enzymes (DNA polymerase and ligases) derived from thermophages have different applications in molecular biology/biotechnology: to generate DNA libraries and develop diagnostics for human and animal pathogens. Furthermore, coat proteins from thermophages are being explored to develop virus-like particle platforms with versatile applications in human and animal health. Overall, bacteriophages, especially those that are thermophilic, have a plethora of applications in One Health.

## 1. Introduction

Bacteriophages or phages are viruses that infect bacteria. It is estimated that more than 10^31^ bacteriophages are present on earth; bacteriophages are thus considered to be the most abundant organisms on earth [1,2,3,4,5,6]. They can be identified/isolated from wherever their host bacteria are present; this includes, but is not limited to, the soil, oceans and lakes, humans and animals, and hot springs and hydrothermal vents. Phages can be grouped into three groups based on the temperature at which their hosts bacteria grow. Phages that infect bacteria with an optimum growth temperature of 37 °C are known as mesophilic phages, while those that infect bacteria that grow at temperatures up to 75 °C are referred to as thermophilic phages. Those that infect bacteria that grow at >75 °C are known as hyperthermophilic phages. Thermophilic and hyperthermophilic phages will be referred to in this review as thermophages. Phages in general have a wide range of applications in human, animal, and plant health as well as in food safety.

### 1.1. One Health and the Application of Mesophilic Phages

The food and animal industries are globalized with a supply chain that has greatly enhanced people’s welfare. However, the globalized nature of the supply chain can contribute to the spread of foodborne and zoonotic diseases; *Salmonella*, *Listeria*, *E. coli* O157, Shiga toxin-producing *E. coli* O104:H4, hepatitis A, Norovirus, etc., have been linked to imported foods [7,8,9]. Thus, the world’s population is vulnerable to foodborne pathogens (including those that are resistant to antibiotics) as well as emerging and re-emerging pathogens (reviewed in [10]). For example, the outbreaks of zoonotic diseases such as SARS, bird flu, Ebola, COVID-19, etc., within the last two decades reminds us that a solution to human health problems (for example, zoonotic diseases, foodborne diseases, antibiotic resistance, and the environment) should be cross-species and multi-disciplinary in nature. To tackle this problem, an interdisciplinary concept “One Health” was created, which is a collaborative effort to achieve optimal health outcomes based on the interconnection between humans, animals, food and agriculture, and the environment [11]. Bacteriophages have contributed and continue to contribute towards the efforts of One Health as described below:

(a) Mesophilic phages have been used within the last decades to combat antibiotic resistance in humans and animals [12,13]. Bacterial resistance to antibiotics is on the rise and alternative approaches are needed to control infections (reviewed in [14,15]). Phages have been used successfully to treat antibiotic-resistant bacterial infections such as: *Acinetobacter baumannii* infection in a 68-year-old diabetic patient [16], *Staphylococcus aureus*-associated chronic rhinosinusitis infection [17], and colistin-only or ceftazidime-sensitive *Pseudomonas aeruginosa* infections in patients with septicemia or with an infected aortic graft [18,19]. Phages have also been used to significantly reduce the concentration of bacteria (Salmonella, Campylobacter) in chickens, Staphylococcus in bovine, and Yersinia, Salmonella, and Bordetella in pigs (reviewed in [20,21]); thus, phages can be used as an alternative treatment for bacterial infections (especially those with antibiotic resistance) in humans and animals. Other studies in plants have also shown that phages can be used as an alternative to copper-based pesticides, which can contaminate the environment, to treat/combat antibiotic resistance in pathogenic plants [22,23,24].
(b) Phages can be used in food safety to prevent the contamination of refrigerated and non-refrigerated food by bacteria [13,25,26,27,28]. For example, an *E. coli* phage, *Tequatrovirus* EP01, has the potential to reduce the concentration of *Salmonella Enteritidis*, *E. coli* O157:H7, *E. coli* O114:K90, and *E. coli* O142:K86 in contaminated meat and milk by 2.18–6.55 log_10_ CFU/sample [27]. In addition to this, a lytic enzyme (endolysin, lysin PhiH5) derived from a *Staphylococcus aureus* phage (PhiH5) has the potential to kill *Staphylococcus aureus* in pasteurized milk [29]. Moreover, GBS B30 endolysin from a streptococcus phage has the potential to lyse three *Streptococcus* spp. (*S. agalactiae*, *S. dysgalactiae*, and *S. uberis*) associated with mastitis in cattle [30].
(c) Phage components have been used as platforms (virus-like particles, VLPs) to develop candidate vaccines against infectious agents of humans and animals [31,32,33].
(d) Phages have been used as biomarkers to indirectly assess the quality of drinking water [34,35]. For example, using T7 phage-conjugated magnetic beads, 1 × 10^4^ colony forming units (cfu)/mL of *E. coli* can be detected in drinking water in 2.5 h; additionally, 10 cfu/mL can be detected using the same system after 6 h of sample pre-enrichment [34]. Other assays, non-phage-based, can detect 1 cfu/mL of *E. coli* (but in 12.5 h [36]) and 2 × 10^6^ cfu/mL (in less than 1 h but it is less sensitive [37]). In addition to this, non-phage-based assays require advanced instruments and training.
(e) Phage components (VLPs) can be used as vehicles to target the delivery of cargo (drugs, vaccine adjuvants such as toll-like receptor ligands) to cells (reviewed in [38,39,40]).
(f) VLPs can be loaded with fluorophores for the imaging of cancer and fibrin clots in vivo (reviewed in [41]).

Thus, phages play a critical role in the One Health approach. They can be used to improve the health of humans, animals, and plants (which both humans and animals dependent on); they can also be used to improve the safety of our food as well as to assess the quality of water, in the environment, for human and animal consumption. Despite the importance/potential application of mesophilic phages in One Health, there are some challenges associated with (that limit) their use.

### 1.2. Limitation of Mesophilic Phages

Mesophilic phages or their components (e.g., endolysins, virus-like particles) cannot maintain their integrity and/or function at environmental conditions above or below their optimum growth temperature (of host bacteria) and thus limit their application/use in One Health as described below.

(a) Endolysins (e.g., lysin PhiH5, LysB4, GBS B30 endolysin) derived from mesophilic phages have the potential to lyse bacteria [29,30,42,43]. However, they lose activity or are inactivated at higher temperatures (such as 55–63 °C for 30 min, 72 °C for 15 min, −20 °C for 30 min) and at pH < 4. Thus, such endolysins cannot tolerate food processing conditions such as pasteurization, nor could they be effective (if applied topically) in treating antibiotic-resistant mastitis-causing bacteria (such as *Staphylococcus aureus*, *Streptococcus* spp.) in the field, especially in areas where temperatures rise above 30 °C.
(b) Virus-like particles (VLPs), derived from mesophilic phages and used as platforms to design vaccines against other infectious agents, are not stable at room temperature [44] and thus require refrigeration. Virus-like particles are empty shells derived from the capsid or envelop proteins of viruses [45]. They lack viral genomes and thus, they are not infectious. In addition to phage-derived VLPs, other viral-derived VLPs have been used as vaccines against viruses (human papillomaviruses, hepatitis B virus, and hepatitis A virus) from which the VLPs are derived [46,47,48,49]. In addition to this, they have also been used as platforms to display and enhance the immunogenicity of less-immunogenic peptide antigens from other infectious agents. The display of foreign peptides on the surface of VLPs enhances the immunogenicity of the peptides and offers protection against infection by the infectious agents from which the peptides were derived [32,50,51,52]. However, VLPs derived from mesophilic viruses have some limitations.
  (i) Like most vaccines, VLP-based vaccines require refrigeration, thus limiting their distribution/use in countries with limited cool-chain infrastructure. Excellent examples are COVID-19 vaccines. Although the vaccines are not VLP-based, they still require refrigeration; 100 million doses of the vaccines were rejected in 2021 by poorer nations due to thermostability-/expiration-related issues [53,54]. VLP-based vaccines are likely to face similar challenges during a pandemic. It is also worth mentioning that a refrigeration requirement for vaccines can also lead to the accidental freezing of vaccines (for facilities that have them); for example, it is estimated that ~14–35% of refrigerated vaccines are accidentally exposed to freezing temperatures during transportation/storage, thus decreasing their efficacy [55,56].
  (ii) Pre-existing antibodies, in human and animal populations, against a platform may affect efficacy. Although pre-existing antibodies to phages that infect bacteria that colonize humans/animals (in a natural setting) have not been reported [57], pre-existing antibodies against some phages/vaccine platforms (in experimental studies) have the potential to attenuate efficacy [58]. In a study, in an experimental setting, pre-existing A3R and 676Z phage-specific IgA antibodies in the gut of mice were shown to reduce the bioavailability of the phages in the gut [59]; a reduction in phage bioavailability is likely to adversely affect phage therapy.
  (iii) Lastly, VLPs derived from mesophilic bacteria have a limitation on the quantity of cargo (drugs, vaccine adjuvants such as toll-like receptor ligands, fluorophores, etc.) that can be loaded into the inner core of the VLPs for delivery into the body/cells. Most phage VLPs (MS2, QB, PP7) with icosahedral structures used for cargo delivery are ~28 nm in diameter [39,40,60], thus limiting the quantity of cargo that can be loaded into them.

To circumvent some of these problems, enzymes for food processing and structural proteins for the development of VLP vaccine platforms can be derived from thermophilic/hyperthermophilic phages; the bacteria that they infect are known as thermophilic bacteria.

## 2. Overview of Thermophages

### 2.1. Diversity of Thermophages

Thermophages can be isolated from hot springs, including hot springs on land [61,62] and submarine alkaline freshwater, as well as coastal springs, deep-sea hydrothermal vents [63], compost piles, dairy environments, soil, industrial hot waters, etc. [64]. Regardless of where they are isolated, thermophages differ in their specificity for the bacterial host they infect, genome/particle size, shape, and structure (Table 1). Some thermophages can infect a wide variety of thermophilic bacterial species, whereas others can infect only a limited number of thermophilic bacteria. For example, phages P23–45 and P74–26B can infect *Thermus aquatic*, *Thermus flavus*, *Thermus thermophilus* HB8, and *Thermus* sp. ATCC 31,674 [62], while the filamentous phage, φOH3, seems to infect only the HB8 strain of *Thermus thermophilus* (and not other strains such as HB27, AT62, TMY, etc.) [65].

**Figure 1:**
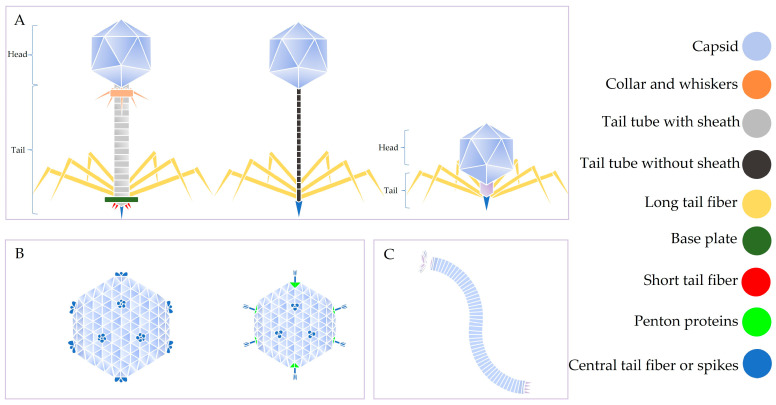
Diversity in the morphology of thermophages. (**A**) Tailed phages, (**B**) tail-less phages, (**C**) filamentous phage. In thermophages that consist of a head and a tail, the tail tube and associated proteins (tail fiber, base plate, etc.) are used for the delivery of the genome to a bacterial cell, while the associated proteins are used for recognition and attachment of the thermophage to bacterial cells, respectively. The tail and associated proteins are not required for the capsid to assemble. The capsid (surrounding the genome) is composed of coat proteins and confers thermostability to thermophages. In some thermophages, the capsid is made up of only one type of coat protein, while in some thermophages, it is made up of more than one coat protein (major and minor capsid proteins). Expression of coat proteins derived from other tailed phages (i.e., mesophilic phages), in an expression vector, in a suitable bacterial host gives rise to VLPs [83].

Thermophage particles come in different shapes (Figure 1); some have an icosahedral capsid (oval head without a tail and are called tail-less phages) [66] while others are oval with a tail (tailed phages) [67]. In addition to tail-less and tailed thermophages, some thermophages are filamentous in shape [65,68,69]. Their genome sizes are also diverse; genome sizes range from 5.2 kb to 152.4 kb [26,65,70], and virion particles are within 60–130 nm wide (for oval-shaped thermophages) to 910 nm long (for filamentous thermophages [69]. Their genomes can be linear [71], circular [72], single-stranded [65], or double-stranded [66,71]. The GC content of the genome ranges from 32 to 68%. Most thermophages infect and replicate in bacteria with optimum growth temperatures from 55 to 70 °C (Table 1).

**Table 1:** Diversity of thermophilic phages.

Phage	Phage Genome	Genome Size (kb)	Morphology	Size of Phage	Isolation Origin	Host Bacteria Isolated from	Optimum/Cultured Temperature of Host Bacteria * (°C)	Optimum/Cultured pH of Host Bacteria	References
P74–26	Double-stranded (ds)DNA	83.32	Oval capsid (head) and a tail	Head: 824 Å Tail: ~800 nm	Kamchatka peninsula hot springs, Russia	*Thermus thermophilus* (*T. thermophilus*)	65	6.0 to 7.0	[73,74]
φOH3	Single-stranded (ss)DNA	5.7	Filamentous	830 nm long and 8 nm wide	Obama hot spring, Japan	*T. thermophilus* HB8	70	7.0	[65]
ϕNS11	dsDNA	57	Oval capsid with spike-like structures	Diameter: 60–75 nm	Beppu hot springs, Japan	*A. acidocaldarius strain* TA6	60 to 65	3.5	[62,64,75]
TP-84	dsDNA	47.7	Oval capsid and a non-contractile tail	Head: 53 × 30 nm Tail: 131 nm long and 3–5 nm wide	Greenhouse soil	*Geobacillus stearothermophilus*	55 to 60	6.5	[64,76]
TS2126	dsDNA	~90	**	**	Hot tap water in Iceland	*Thermus scotoductus*	65	7.5	[77,78]
ΦGVE2	dsDNA	40.9	**	**	Deep-sea hydrothermal fields in the Pacific	*Geobacillus* sp. E263	60 to 65	7.0	[63,64,79]
MMP17	dsDNA	33.5 to 39.5	Oval capsid and a tail	Head: 42 nm Tail: 120 nm long and 17 nm wide	Eryuan hot spring, China	*Meiothermus*	55 to 60	6.0 to 7.0	[80]
P23–77	dsDNA	17.04	Icosahedral capsid with spikes on the vertices, and an internal lipid membrane, no tail	Diameter: 78 nm	Alkaline hot springs, New Zealand	*T. thermophilus*	70	6.0 to 7.0	[72]
ΦIN93	dsDNA	19.6	Icosahedral	Diameter: ~130 nm	Hot spring soil, Japan	*Thermus aquaticus*	70	6.0 to 7.0	[61]
PH75	ssDNA	6.5	Filamentous	Length: 910 ± 17 nm	Hot spring, New Zealand	*T. thermophilus*	70	8.3	[62,69]
ϕTMA	dsDNA	151.5	Icosahedral capsid and a contractile tail	Head: ~125 nm Tail: ~175 nm	Atagawa hot spring, Japan	*T. thermophilus*	65	6.0 to 7.0	[26]
ϕYS40	dsDNA	152.4	Oval capsid and a contractile tail	Head: 125 nm Tail: 178 nm long and 27 nm wide	Atagawa hot spring, Japan	*T. thermophilus*	65	6.0 to 7.0	[26,81,82]
RM378	dsDNA	129.9	Isometric capsid and a tail	Head: 85–95 nm Tail: 150 nm	Slightly saline, geothermal environments, and hot springs, Iceland	*Rhodothermus marinus*	65	7.2	[70]

* They can also grow at higher temperatures but at lower rate; ** No information available.

### 2.2. Features That Confer Thermostability in Extreme Hot Environments

(a) The capsid: The stability of the capsid of thermophages is crucial for their survival in host bacteria, as well as in the environment, following their release from the host (i.e., until they infect the next bacteria). The organization/geometry of capsomeres (coat proteins), which make up the capsid, contributes to the stability of the capsid. It is believed that the capsid of thermophage P74–26 is stable at 80 °C (unlike that of a mesophilic lambda phage heated at the same temperature) due to the fact that its coat proteins are topologically “tied” together by lassos [74]. The thermostability of the capsid thus protects the genome from environmental stress such as high temperature and low/high pH. For example, thermophage ϕNS11 (PhiNS11) remains stable at 60 °C (pH 2–5), while its naked DNA is degraded at 55 °C (pH 4) [84]; ϕNS11 infects acidophilic thermophilic bacteria and thus, it also considered to be an acidophilic phage.
(b) The guanine–cytosine (GC) content: The GC content of nucleic acids of thermophages also contributes to the thermostability of their genomes at high temperatures. For example, the GC content of thermophage P23–77 and ΦIN93 is 68% and 66%, respectively [66,85], while that of mesophilic phages (e.g., *Salmonella* phage BIS20 and *E. coli* lambda phage) is 53% and 50%, respectively [86,87]. Given its higher GC contents, higher temperatures are required to break the three hydrogen bonds between guanine and cytosine than the two hydrogen bonds between thymine and adenine in mesophilic phages. This thus enhances the thermostability of genomes of thermophages compared to those of mesophilic phages.
(c) Osmolarity: Inorganic ions (calcium, Ca^2+^, or potassium, K^+^) in salts enhance the stability of thermophages (the head, tail, and DNA). Positive ions help neutralize negative phosphate charges on sugar molecules (on nucleic acid) that repel each other, thus stabilizing DNA. As a matter of fact, the supplementation of thermophage (e.g., TP-84) growth media with calcium or potassium ions enhances survival/titers by 18–10,000-fold at 65 °C [76,88]. On the contrary, the chelation of these ions with EDTA (ethylenediaminetetraacetic acid) or the addition of phosphate (additional negative ions that promote repulsion) leads to the dissociation of the tail from the head of thermophage TP-84 [89].

Overall, the osmolarity of a host bacterium and its environment, the GC content of thermophages, and the geometry/tethering of capsomeres on the capsid all contribute to the stability of thermophages and, subsequently, their ability to thrive at high temperatures.

## 3. Application of Thermophages in One Health

While live thermophages cannot be used to control bacterial infections in humans, animals, or pathogenic plants (due to their inability to infect/replicate in bacteria that infect humans, animals, and plants), their components (enzymes, capsid proteins) can be used in molecular biology to improve the health of humans and animals, as well as in food safety. As mentioned above, components, such as endolysins and virus-like particles, derived from mesophilic phages lose activity (with time) at room temperature and above; this thus limits their application in food processing, in treating antibiotic-resistant mastitis-causing bacteria in the field, and in treating pathogenic plants, as well as their applications as platforms for vaccine design. These limitations can be overcome by using enzymes (endolysins and viral polymerases) and coat proteins, derived from thermophages.

### 3.1. Thermophage Enzymes in Molecular Biology

Enzymes derived from thermophages have a lot of applications in molecular biology. For example, PyroPhage 3173 DNA Pol polymerase used in reverse transcription (RT) polymerase chain reaction (PCR) was derived from a thermophage genomic library generated using samples collected from Yellowstone hot springs [90]. RT-PCR/PCR is normally performed using a two-enzyme system: a reverse transcriptase derived from MMLV (Moloney Murine Leukemia Virus) and *Taq* polymerase derived from thermophilic bacteria (*Thermus aquaticus*). While MMLV RT can convert RNA to cDNA for PCR, it lacks thermostability and thus, *Taq* polymerase, which is active at temperatures up to 95 °C for 40 min (reviewed in [91]), is used as an enzyme for PCR after reverse transcription. With the expression and purification of PyroPhage 3173 DNA Pol polymerase (thermostable at 94 °C for 11 min) from thermophages, RT-PCR/PCR can now be performed using a single enzyme. The enzyme catalyzes both reverse transcription and DNA amplification in a single reaction [90,92]. A modified version of PyroPhage 3173 DNA Pol polymerase, OmniAmp Pol, has been developed and used in point of care (POC) diagnostic tests in LAMP (loop-mediated isothermal amplification) assays to detect human and animal infectious agents such as swine influenza virus, porcine circovirus-2, West Nile virus, Ebola virus, Crimean–Congo hemorrhagic fever, Bovine viral diarrhea virus, *Edwardsiella ictaluri*, *Bacillus atrophaeus,* and *Staphylococcus aureus*. It is worth mentioning that the POC LAMP assays using OmniAmp Pol amplified target genes in <30 min compared to RT-LAMP using *Bst* polymerase (which takes about 20% longer) [93].

In addition to the above enzymes, another thermophage enzyme (Phage TS2126 ligase) isolated from a thermophage TS2126 [77] is very crucial in molecular biology; the enzyme is stable at 60–65 °C. Phage TS2126 ligase (marketed as CirLigase^TM^) is used to circularize single-stranded DNA for many applications (rolling-circle replication/transcription) [94]. The enzyme has been used to generate DNA libraries for next-generation sequencing [95]. In addition to Phage TS2126 ligase, RM378 RNA ligase 1 (derived from thermophage RM378) ligates both ssDNA and RNA molecules. Given its thermostability features (optimum temperature 60–64 °C), it is used in the RNA-ligase-mediated rapid amplification of cDNA ends [96], and the adenylation of small RNA sequencing adapters [97]. Thus, phage TS2126 ligase, PyroPhage 3173 DNA Pol polymerase, and its modified version, OmniAmp Pol, have the potential to make a significant contribution to One Health, especially in human and animal health. The enzymes can contribute to the sequencing of the genomes of humans and animals. They can also contribute towards the sequencing of their microbiome, thus contributing to the diagnosis of new pathogens with unknown treatment.

### 3.2. Thermophage Enzymes in Veterinary Medicine

Enzymes isolated from different thermophages are stable at high temperatures and therefore have potential applications in veterinary medicine and One Health in general. For example, an endolysin (ΦGVE2 amidase) isolated from a deep-sea thermophilic bacteriophage, Geobacillus virus E2 (ΦGVE2), is thermostable at temperatures up to 80 °C. The fusion of the endolysin with an endolysin from a *Clostridium perfringens* phage (recombinant fusion endolysin called PlyGVE2CpCWB) lyses *Clostridium perfringens* [98]. *Clostridium perfringens* is a foodborne pathogen (infects both humans and animals) and is associated with diarrhea in humans, cattle, horses, pigs, chickens, goats, etc. [99,100]; thus, it is present in fecal matter and in soil, where it can form endospores (resistant to environmental stress). Given the stability/activity of PlyGVE2CpCWB endolysin (95% activity after a 30 min incubation at 50 °C), the enzyme can be added to animal feed/water or used in animal facilities to prevent contamination with *Clostridium perfringens*; it can also be used in food processing to prevent the contamination of chicken/meat with the bacteria.

Recently, another thermostable endolysin (MMPphg), derived from thermophage MMP17, has been expressed and purified [101]. MMPphg is thermostable at temperatures up to 65 °C for 30 min (80% of its activity retained). Unlike other endolysins which are efficacious mostly against Gram-positive bacteria (reviewed in [102]), MMPphg has antimicrobial activities against both Gram-negative and Gram-positive bacteria including antibiotic-resistant strains. At 37 °C for 1 h, the enzyme lysed *Escherichia coli* O157, *Staphylococcus aureus* (including resistant strain KMUST1606BL1486), *Salmonella enterica* serovar Enteritidis, *Salmonella enterica* serovar Typhi, *Salmonella enterica* serovar Paratyphi B, *Shigella dysenteriae,* and *Klebsiella pneumoniae* (this includes nine antibiotic-resistant strains). Bacterial cells in antimicrobial assays were reduced by 0.96–3.4 log_10_ [101]. Given the broad spectrum of antimicrobial activity and the thermostability of endolysin MMPphg, the enzyme could be used to treat *Staphylococcus aureus* infections associated with bovine mastitis.

### 3.3. Thermophage Coat Proteins and Potentials for Vaccine Platforms

Vaccines play a crucial role in protecting the health of humans and animals, making them an essential component of One Health. Coat proteins derived from thermophages can serve as an alternative source of structural proteins to develop virus-like particle platforms for vaccine design (i.e., display antigens from human and animal infectious agents) and to deliver cargo into the body/cells. P23–77 and ΦIN93 are thermophages with coat proteins with great potential to develop VLP platforms for vaccine design. P23–77 and ΦIN93 were isolated from thermophilic bacteria with an optimum growth temperature of 70 °C [61,72]. P23–77 has a diameter of 78 nm, while ΦIN93 has a diameter of 130 nm. The GC contents of the capsid proteins are very high (~66%); the coat proteins of P23–77 are thermostable at a temperature > 80 °C [103,104], and its capsid is composed of 6 nm-thick multilayered proteins [72]. Given the aforementioned features, VLPs derived from these two thermophages (P23–77 and ΦIN93) are likely thermostable at high temperatures, and their inner cores can accommodate large quantities of cargo.

Studies have shown that the capsid of P23–77 consists of two major coat proteins (VP16 and VP17) and one minor coat protein (VP11) [66,104]; VP11 is a ~22 KD protein, VP16 is ~19 KD, and VP17 is a ~32 KD protein. It is estimated that the viral capsid of P23–77 is assembled from 147 copies of VP11, 1080 copies of VP16, and 540 copies of VP17 [103,104]. The capsid protein of ΦIN93, on the other hand, is believed to be composed of two putative coat proteins (ORF13 and ORF14) based on sequence homology with the VP16 and VP17 of P23–77. ORF13 has 80% sequence identity with VP16, while ORF14 has 73% sequence identity with the VP17 of P23–77; additionally, the structures of coat proteins from both viruses are similar [61,105]. A sequence in thermophage ΦIN93 that is homologous to VP11 (minor capsid protein of P23–77) has not been identified, suggesting that the capsid protein of ΦIN93 is composed of only two coat proteins, ORF13 and ORF14. Attempts have been made within the last few years to assess the potential of coat proteins from P23–77 [104] and ΦIN93 [61,105] to assemble into VLPs. Pawlowski et al. [104] expressed and purified (separately from *E. coli*) VP11, VP16, and VP17 coat proteins; a mixture of the three purified coat proteins showed that they can form complexes in vitro. More recently, in a PhD dissertation, Yadav [106] successfully co-expressed (for the first time) truncated versions of VP16 and VP17 coat proteins together with VP11; co-expressed proteins formed concentric cycles, but their diameters were less than 70 nm. Recently, Zhai et al. [105] co-expressed ORF13 and OFR14 coat proteins of ΦIN93 (full-length and truncated versions) in *E. coli*. Similar to those of VP11, VP16, and VP17, the co-expression of the truncated versions of ORF13 and OFR14 formed oval structures with diameters (~75 nm to ~100 nm) smaller than those of the authentic ΦIN93 virus (130 nm). More studies are required to authenticate these oval structures and to further assess whether other structural proteins are required for the coat proteins to form complexes or assemble into VLPs.

## 4. Conclusions and Perspectives for the Future

Bacteriophages, especially their components (lytic enzymes and coat proteins), have a plethora of applications in One Health. Mesophilic phages can be used to combat antibiotic-resistant bacteria in humans, animals, and plants, and also in food processing (especially vegetables that may be contaminated with fecal water). In addition to live phages, lytic enzymes, especially those derived from thermophages (with greater thermostability), can be used in food processing, in treating water for livestock that may be contaminated with pathogenic bacteria, and in treating bacteria-associated mastitis in cattle. Temperature is very critical in the livestock industry; mesophilic phage components may present challenges for development and utilization in high-temperature environments, which is relatively common in animal rearing and product processing. While a lot of studies have demonstrated the efficacy of thermophilic endolysins (e.g., MMPphg, ΦGVE2 amidase) against a select group of bacteria (including *Escherichia coli* O157, *Staphylococcus aureus*, *Clostridium perfringens*, *Salmonella enterica*, *Klebsiella pneumoniae*, etc.) [98,101], more studies are required to assess their efficacy against other bacteria of veterinary importance such as *Streptococcus* infections (associated with bovine mastitis), *Bacillus anthracis* (associated with anthrax and forms endospores), etc. In addition to this, the ability of endolysin MMPphg to control *Paenibacillus larvae* (especially its spores) among other pathogenic honeybee bacteria should also be assessed. *Paenibacillus larvae* is a Gram-positive bacterium associated with fatal disease in the larvae of honeybees; it is present worldwide, with a high prevalence in the US and Europe [107,108]. While an endolysin (PlyPL123) isolated from a mesophilic phage (phiIBB_Pl23) lyses the bacterium (without toxicity to larvae from bees), the endolysin loses activity within a week at room temperature [109]. Thus, PlyPL123 endolysin may not be applicable in the field, especially where temperatures rise above 25 °C. An effective thermostable endolysin that is appliable in the field is needed to prevent the use of antibiotics in honeybee production, thus preventing its introduction into humans. Thermophage components have promising application prospects in the field of human and veterinary health.

Coat proteins derived from thermophages also have a lot of applications in One Health. They can be used to develop thermostable vaccines, to deliver cargo to specific cells, and for in vivo imaging. If successfully developed, VLPs, especially from ΦIN93 (with a diameter of 130 nm), will have the capacity to be loaded with more cargo (vaccine adjuvants, imaging fluorophores) for targeted delivery to cells. The diameter of ΦIN93 is almost twice the size of thermophilic P23–77 (78 nm) and more than four times the size of mesophilic MS2 VLPs (~28 nm), which is widely used in the field to deliver cargo [110,111,112]. The sizes of P23–77 and ΦIN93 have additional benefits as platforms for vaccine design/immunization. Studies have shown that nanoparticles less than 200 nm, such as VLPs, can directly enter the lymphatic vessels from the site of immunization (without association with injection-site antigen-presenting cells) and drain freely to the lymph nodes [113,114]. With this in mind, VLPs derived from P23–77 and ΦIN93 are likely to enter directly into the lymphatic vessels and drain efficiently to lymph nodes. Another benefit of VLPs derived from P23–77 and ΦIN93 is the fact that the public, including animals, lack pre-existing antibodies against these thermophages. Thus, VLPs derived from P23–77 and ΦIN93 are likely to be highly immunogenic in humans and animals. They can therefore be used to display foreign peptides on their surfaces, thus enhancing the immunogenicity of the peptides.

While thermophilic endolysins and their coat proteins have enormous potential in One Health, some challenges lie ahead. For example, the application of endolysins to treat gut bacterial infections in humans and animals may be limited, given challenges to deliver them to the gut without inactivation by gastric juice, which is highly acidic. To overcome this challenge, endolysins from a double extremophile ϕNS11 should be explored. As mentioned above, ϕNS11 infects a thermophilic bacterium that grows in an acidic environment (pH 3) [64,115]. Thus, endolysins derived from this phage are likely to resist the pH of gastric juice. Furthermore, it is not clear whether bacteria will develop resistance, in the near future, to endolysins, as they have to antibiotics. Additionally, the efficacy of ϕNS11 endolysins against endospores also needs to be explored.

## Data Availability

Not applicable.
